# Supplementary material for: SDE2 integrates into the TIMELESS-TIPIN complex to protect stalled replication forks
Source: Nat Commun. 2020 Oct 30;11:5495. doi: 10.1038/s41467-020-19162-5 (PMC7603486; doi:10.1038/s41467-020-19162-5)
Supplement: Supplementary file 1 — Supplementary Information [file 41467_2020_19162_MOESM1_ESM.pdf]

Supplementary Information

SDE2 Integrates into the TIMELESS-TIPIN Complex to Protect Stalled Replication Forks

Julie Rageul[1,6], Jennifer J. Park[1,6], Ping Ping Zeng[1], Eun-A Lee[2], Jihyeon Yang[2], Sunyoung Hwang[2], Natalie Lo[1], Alexandra S. Weinheimer[3], Orlando D. Schärer[2,4], Jung-Eun Yeo[2]*, and Hyungjin Kim[1,5]*

[1]Department of Pharmacological Sciences, State University of New York at Stony Brook, Stony Brook, New York 11794, USA
[2]Center for Genomic Integrity, Institute for Basic Science, Ulsan National Institute of Science and Technology, Ulsan 44919, Republic of Korea
[3]Department of Biochemistry and Cell Biology, State University of New York at Stony Brook, Stony Brook, New York 11794, USA
[4]Department of Biological Sciences, School of Life Sciences, Ulsan National Institute of Science and Technology, Ulsan 44919, Republic of Korea
[5]Stony Brook Cancer Center, Renaissance School of Medicine at Stony Brook University, Stony Brook, New York 11794, USA
[6]These authors contributed equally

* Correspondence:

hyungjin.kim@stonybrook.edu, jyeo@ibs.re.kr

Supplementary Fig. 1

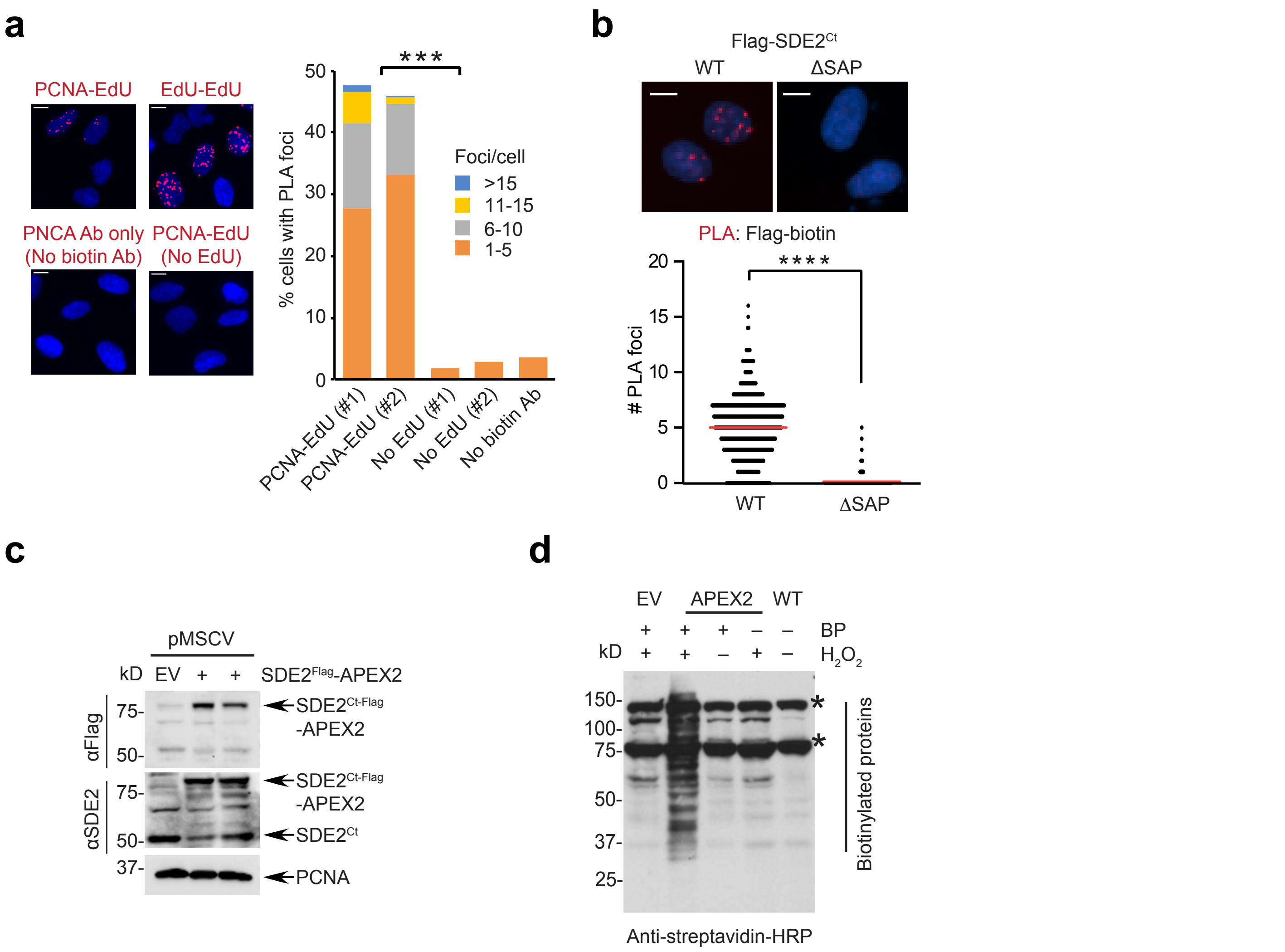

Supplementary Figure 1.
(related to Figure 1).

a Left: representative images of controls for the SDE2:EdU-biotin PLA assay. PCNA:EdU PLA foci were from U2OS cells pulsed for 8 min with DMSO or 125 µM EdU. Either biotin antibody or EdU incubation is omitted as negative controls. Scale bar, 10 µm. Right: quantification of cells positive for PCNA:EdU PLA foci and number of foci per cell (>400 cells per condition, ****P*<0.001, Student's t-test on means of duplicates). **b** Top: representative images of PLA foci between Flag-SDE2^{Ct} and EdU. EdU-incubated U2OS cells expressing N-terminal UBL-deleted Flag-SDE2 wild-type (WT) or ΔSAP mutant (aa385-451) were fixed, and PLA foci were visualized by anti-Flag and anti-biotin antibodies. Bottom: dot plot showing PLA foci numbers within Flag:biotin PLA positive cells. Red bars represent the mean (n=2 biologically independent experiments, a representative experiment is shown, *****P*<0.0001, Mann-Whitney test). Scale bar, 10 µm**.** **c** Western blot (WB) analysis of U2OS cells stably expressing SDE2-APEX2 by retroviral transduction (vs. pMSCV empty vector, EV). Cells infected with two different titers are shown. **d** Visualization of SDE2-APEX2-mediated proximity biotin labeling of endogenous proteins by streptavidin-horseradish peroxidase (HRP) Western blotting. Negative controls in which BP, H_2O_2, or SDE2-APEX2 were omitted are shown. Asterisks indicate endogenous biotinylated proteins known to migrate near 130, 75, and 72 kD.

Supplementary Fig. 2

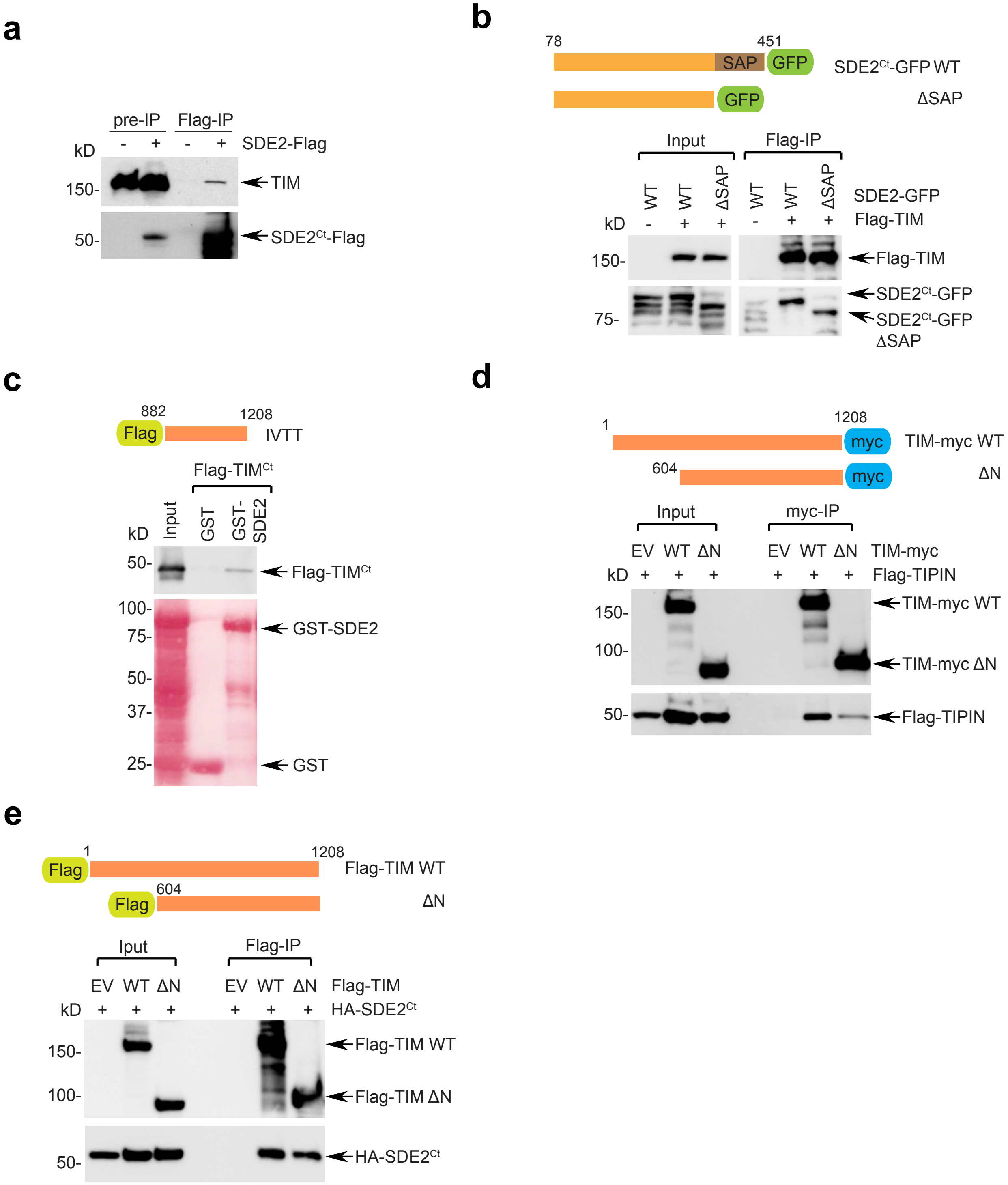

Supplementary Figure 2.

(related to Figure 2).

a Anti-Flag co-immunoprecipitation (IP) of SDE2-Flag with endogenous TIM in U2OS cells. (-: empty vector). **b** Anti-Flag co-IP of Flag-TIM with SDE2-GFP WT, or ΔSAP (Δ385-451) mutant in 293T cells. Schematic of the SDE2-GFP construct variants is shown above. **c** GST pull-down of *in vitro* transcribed and translated (IVTT) TIM^{Ct} (aa882-1208) with purified GST or GST-SDE2. **d** Anti-myc co-IP of TIM-myc WT or Δ1-603 (ΔN) mutant with Flag-TIPIN in 293T cells. Schematic of the TIM-myc construct variants is shown above. **e** Anti-Flag co-IP of Flag-TIM WT or ΔN mutant with $HA\text{-}SDE2^{Ct}$ in 293T cells.

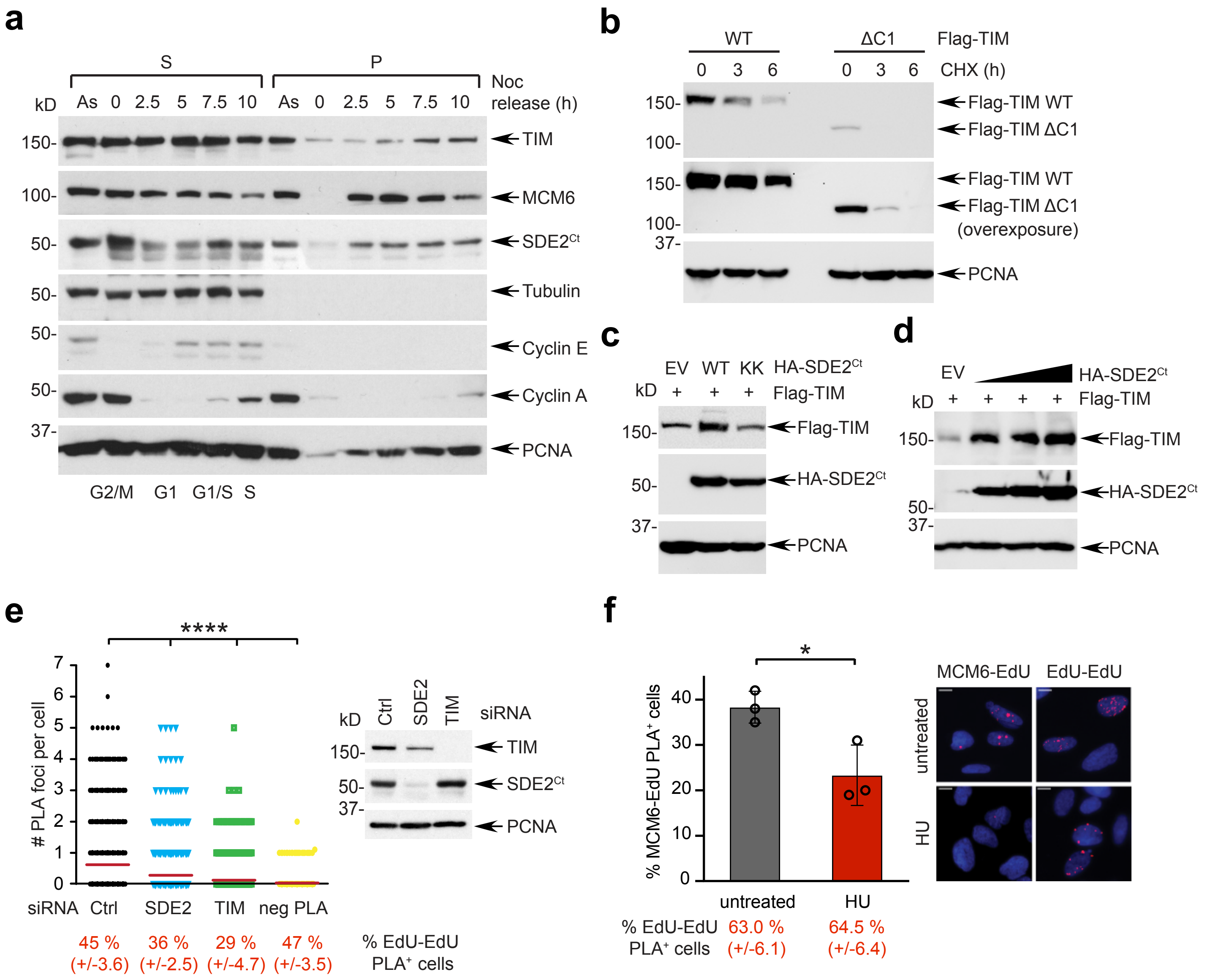
a
S
P
Noc release (h)
kD
As 0 2.5 5 7.5 10 As 0 2.5 5 7.5 10
150-
TIM
100-
MCM6
50-
SDE2Ct
50-
Tubulin
50-
Cyclin E
50-
Cyclin A
37-
PCNA
G2/M G1 G1/S S
b
WT ΔC1 Flag-TIM
0 3 6 0 3 6 CHX (h)
150-
100-
Flag-TIM WT
Flag-TIM ΔC1
150-
100-
37-
Flag-TIM WT
Flag-TIM ΔC1
(overexposure)
PCNA
c
EV WT KK HA-SDE2Ct
kD + + + Flag-TIM
150-
Flag-TIM
50-
HA-SDE2Ct
37-
PCNA
d
EV HA-SDE2Ct
kD + + + + Flag-TIM
150-
Flag-TIM
50-
37-
HA-SDE2Ct
PCNA
e
****
PLA foci per cell
0 1 2 3 4 5 6 7
siRNA Ctrl SDE2 TIM neg PLA
45 % (+/-3.6) 36 % (+/-2.5) 29 % (+/-4.7) 47 % (+/-3.5)
% EdU-EdU PLA+ cells
kD Ctrl SDE2 TIM siRNA
150-
TIM
50-
37-
SDE2Ct
PCNA
f
*
% MCM6-EdU PLA+ cells
0 10 20 30 40
untreated HU
% EdU-EdU PLA+ cells
63.0 % (+/-6.1)
64.5 % (+/-6.4)
MCM6-EdU EdU-EdU
untreated
HU

Supplementary Figure 3.
(related to Figure 3).

a Cell cycle-dependent changes of TIM and $SDE2^{Ct}$ levels in both S and P fractions. U2OS cells were synchronized at the G2/M boundary with 100 ng/mL nocodazole and released into fresh medium to let cells traverse from G1 to S phase. Cells were harvested at the indicated times and fractionated into S and P fractions, followed by WB analysis. **b** Comparison of degradation kinetics of Flag-TIM WT or ΔC1 upon treatment of 100 µg/mL CHX for the indicated times. **c** WB analysis of 293T cells expressing Flag-TIM, transfected with either HA-$SDE2^{Ct}$ WT or KK (K132A/K135A) mutant. **d** Elevation of cellular Flag-TIM levels in 293T cells in response to increasing amount of HA-$SDE2^{Ct}$. **e** Left: quantification of TIM:EdU PLA foci numbers from PLA-positive cells. The percentage of EdU:EdU PLA positive cells is shown. The number of foci per positive cells was pooled from three independent experiments (*****P*<0.0001, Mann-Whitney test). Right: WB analysis to confirm knockdown efficiency. **f** Left: Quantification of cells positive for MCM6:EdU PLA foci (as percentage of total). Percentages of EdU-EdU PLA positive cells are also shown for each experimental condition (n=3 from two biologically independent experiments, >500 cells per replicate, **P*<0.05). Right: representative images of MCM6:EdU PLA foci from U2OS cells pulsed with EdU and either directly fixed (untreated) or treated for 1 h with 500 µM HU before fixation. Scale bar, 10 µm.

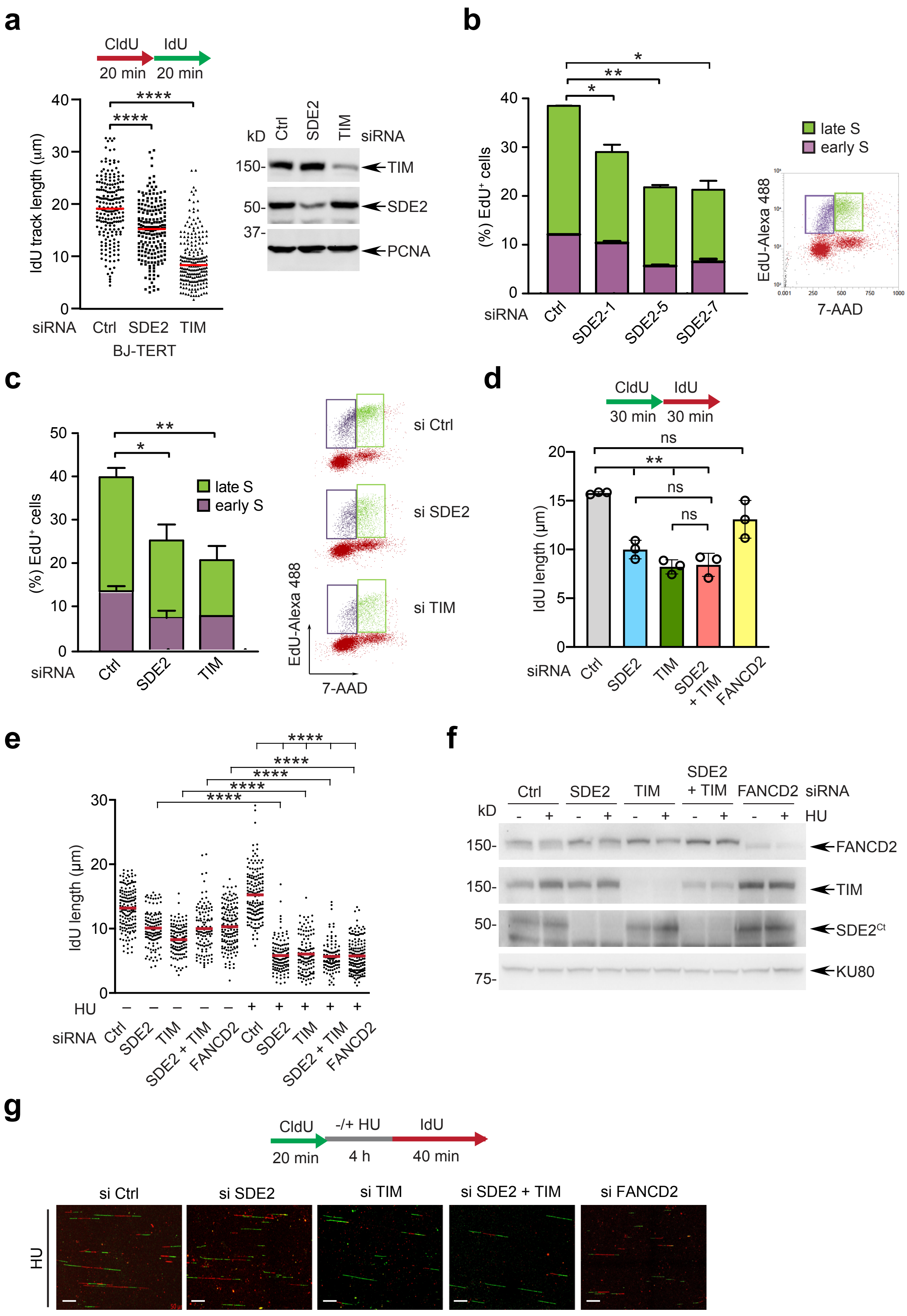
a
CldU IdU
20 min 20 min
IdU track length (μm)
****
****
siRNA Ctrl SDE2 TIM
BJ-TERT
kD Ctrl SDE2 TIM siRNA
150- TIM
50- SDE2
37- PCNA
b
(%) EdU⁺ cells
*
**
*
siRNA Ctrl SDE2-1 SDE2-5 SDE2-7
late S
early S
EdU-Alexa 488
7-AAD
c
(%) EdU⁺ cells
**
*
late S
early S
siRNA Ctrl SDE2 TIM
si Ctrl
si SDE2
si TIM
EdU-Alexa 488
7-AAD
d
CldU IdU
30 min 30 min
IdU length (μm)
ns
**
ns
ns
siRNA Ctrl SDE2 TIM SDE2 + TIM FANCD2
e
****
****
****
****
****
IdU length (μm)
HU – – – – – + + + + +
siRNA Ctrl SDE2 TIM SDE2 + TIM FANCD2 Ctrl SDE2 TIM SDE2 + TIM FANCD2
f
Ctrl SDE2 TIM SDE2 + TIM FANCD2 siRNA
kD - + - + - + - + - + HU
150- FANCD2
150- TIM
50- SDE2Ct
75- KU80
g
CldU -/+ HU IdU
20 min 4 h 40 min
si Ctrl si SDE2 si TIM si SDE2 + TIM si FANCD2
HU

Supplementary Figure 4.

(related to Figure 4).

a Immortalized BJ-hTERT cells were knocked-down for SDE2 or TIM and labeled with CldU and IdU. Left: Representative dot plot of the IdU track length from two independent experiments (>190 tracks per condition, n=2, *****P*<0.0001, Mann-Whitney). Right: WB to confirm the knockdown. **b** Left: EdU cell cycle analysis of U2OS cells transfected with independent SDE2 siRNA oligos. (n=2, mean ± SD, ***P*<0.01, **P*<0.05, Student's t-test, for the comparison of late S phase cells). Right: a representative FACS plot defining early and late S phases from EdU-positive cells **c** EdU cell cycle analysis of U2OS cells transfected with the indicated siRNAs (n=3 biologically independent experiments, mean ± SD, ***P*<0.01, **P*<0.05, Student's t-test, for the comparison of late S phase cells). Representative FACS plots showing the distribution and percentage of EdU-positive cells in early and late S phases are shown. **d** Mean IdU DNA fiber track lengths from U2OS cells transfected with indicated siRNAs from Fig. 4e (n=3 biologically independent experiments, mean ± SD, ***P*<0.01, Student's t-test, ns, not significant). **e** Dot plot of the CldU and IdU track lengths to measure replication fork restart speed (i.e. delayed start) after HU-mediated fork stalling in U2OS cells transfected with the indicated siRNAs from Fig. 4f. A representative result from three independent experiments is shown (n>150 fibers analyzed, *****P*<0.0001, Mann-Whitney). **f** Western blotting to confirm the knockdown. **g** Representative images of DNA fiber tracks before and after HU-mediated fork stalling. Increase of green track-only DNA fibers represents increased fork stalling. Scale bar, 10 μm.

Supplementary Fig. 5

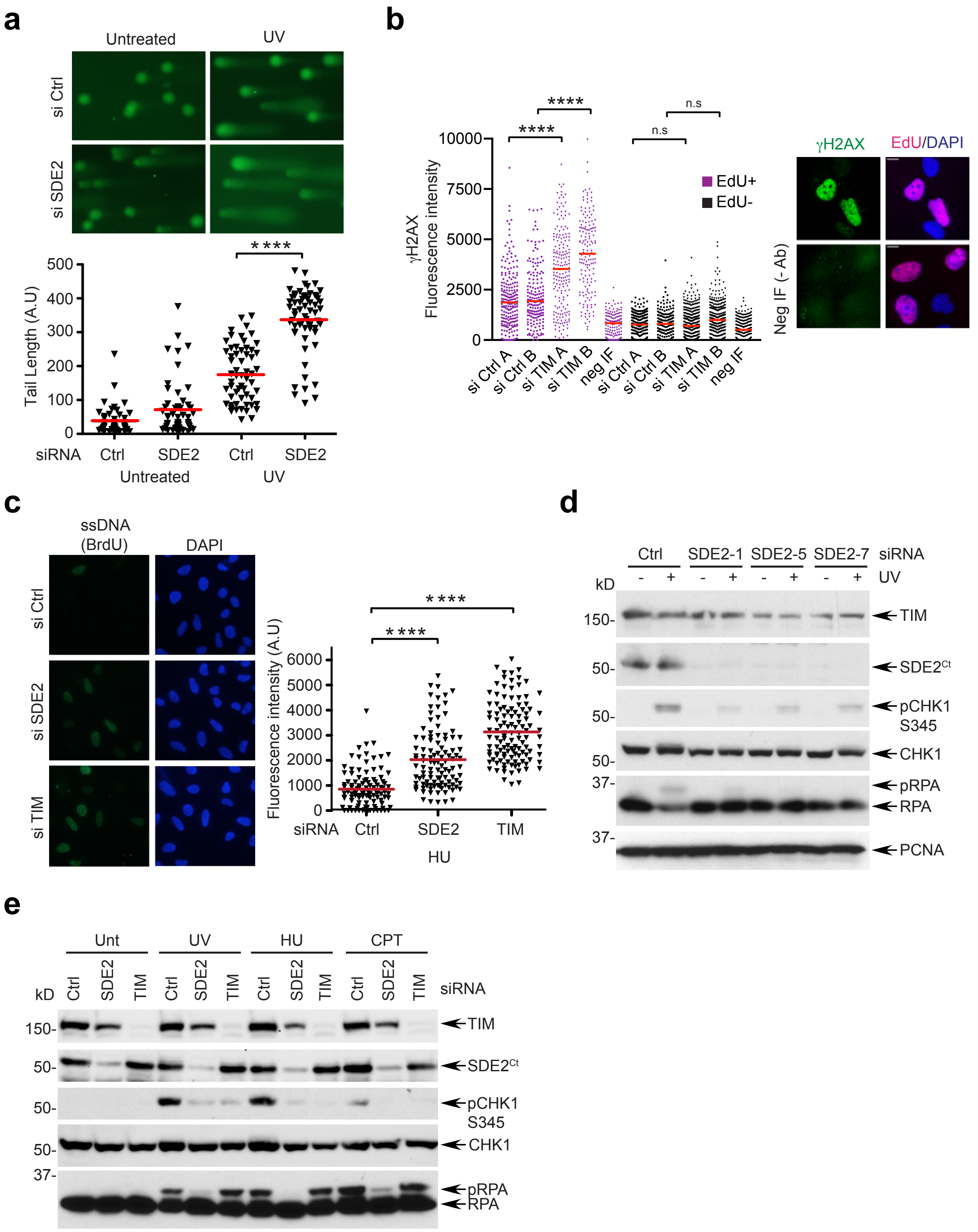

Supplementary Figure 5.
(related to Figure 5).

a Top: representative images of DNA comets from siRNA-transfected U2OS cells 4 h after irradiation with 40 J/m^2 UVC. Bottom: quantification of DNA comet tail lengths from two independent experiments. Red bars represent median (>50 nuclei per condition, ****P<0.0001, Mann-Whitney test). **b** Left: representative dot plot of γH2AX fluorescence intensities in EdU positive versus negative cells from two independent experiments. U2OS cells were first labeled for 30 min with EdU and then treated with 2 mM HU for 4 h, followed by click reaction with Alexa Fluor 647-azide. siRNA A and B are technical replicates. Red bars represent the medians (>140 nuclei per replicate, n=2, ****P<0.0001, Mann-Whitney test). Right: representative images of γH2AX foci and EdU double staining. **c** Left: representative images of native BrdU staining after treatment with 2 mM HU for 4 h in U2OS cells transfected with the indicated siRNAs. Cells were pre-treated with 10 μM BrdU for 48 h before irradiation to mark single-stranded DNA. Right: quantification of BrdU staining. Red bars represent median (>100 nuclei per condition, n=2 biologically independent experiments, ****P<0.0001, Mann-Whitney test). **d** CHK1 phosphorylation at S345 4 h after irradiation with 40 J/m^2 UVC in U2OS cells transfected with independent SDE2 siRNA oligos. **e** CHK1 phosphorylation after the indicated types of DNA damages (2 mM HU for 4 h, 4 h after 40 J/m^2 UVC irradiation, or 100 nM camptothecin (CPT) for 4 h) in U2OS cells transfected with the indicated siRNAs.

Supplementary Fig. 6

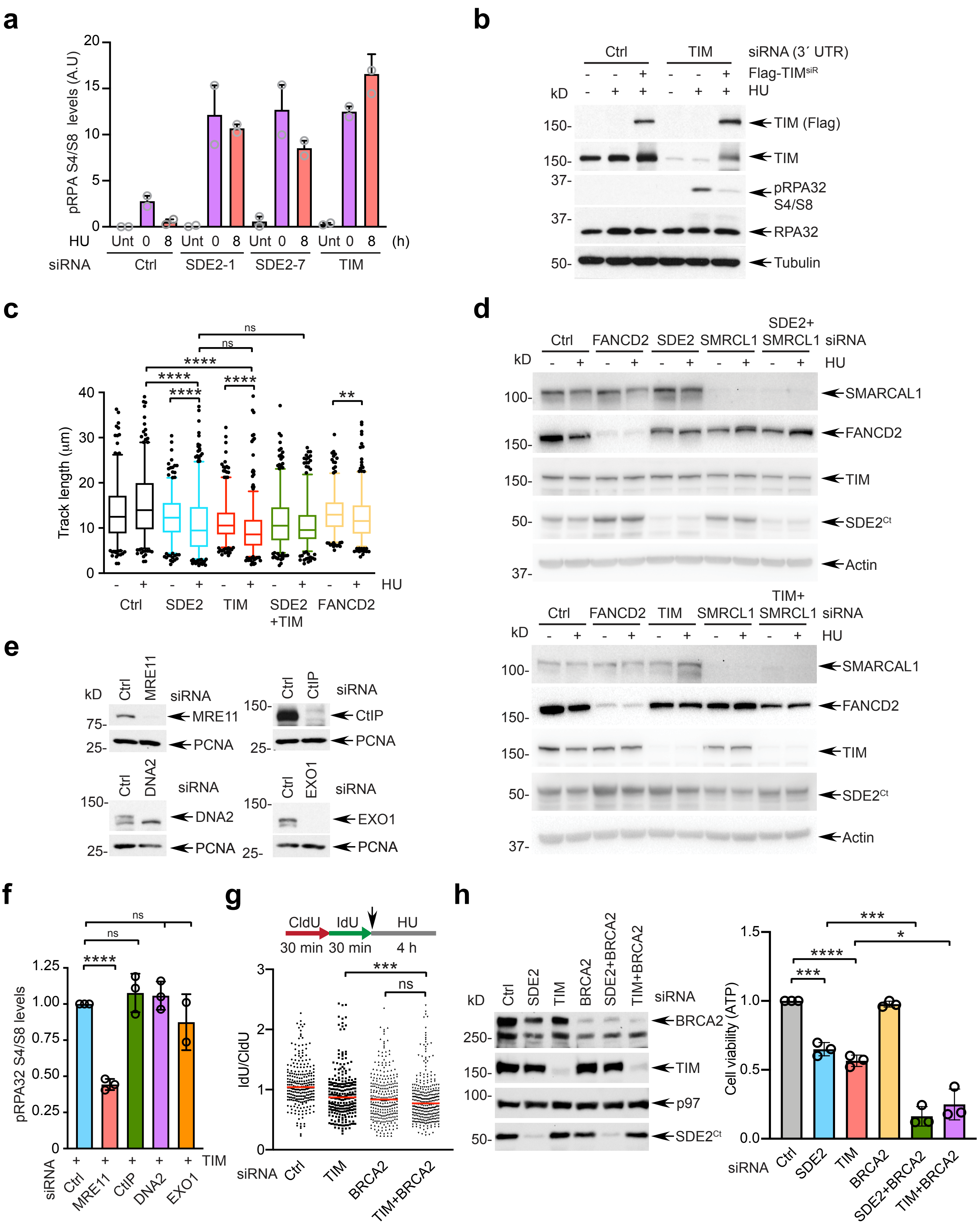

Supplementary Figure 6.
(related to Figure 6).

a Quantification of pRPA32 S4/S8 levels in siRNA-transfected U2OS cells recovered from the overnight treatment of 250 μM HU. Mean from two biologically independent experiments is shown. **b** Rescue of the elevated pRPA32 S4/S8 phenotype. U2OS cells were serially transfected with siRNA TIM that targets the 3´ UTR of *TIM* mRNA and Flag-TIM encoding plasmid. Cells were treated with 250 μM HU for 20 h and recovered for 4 h in fresh medium to assess pRPA32 S4/S8 levels. **c** Box plots of CldU track lengths from siRNA-transfected U2OS cells untreated (unt) or treated with 2 mM HU for 4 h. FANCD2 knockdown serves as a positive control showing impaired fork protection. (n=3 biologically independent experiments, line in the box=median; box=25th and 75th percentiles; bar=5th and 95th percentiles, *****P*<0.0001, ***P*<0.01, ns, not significant, Mann-Whitney test). **d** WB analysis to confirm knockdown of SDE2 and TIM individually or together with SMARCAL1 in U2OS cells, used for DNA combing analysis. **e** WB analysis to confirm siRNA knockdown of individual nucleases. **f** Quantification of pRPA32 S4/S8 levels in U2OS cells co-depleted of TIM and individual nucleases upon recovery from overnight treatment of 250 μM HU (n=3 biologically independent experiments, mean ± SD, *****P*<0.0001, Student's t-test). **g** Dot plot of DNA fiber IdU/CldU track length ratios from U2OS cells either knocked-down with TIM only or TIM and BRCA2 together. A representative plot is shown from three biologically independent experiments (****P*<0.001, Mann-Whitney). **h** Left: WB analysis to confirm the knockdown of SDE2 and TIM individually or in combination with BRCA2. Right: luminescence-based ATP viability assay of U2OS cells transfected with the indicated siRNAs. Measurement was performed 7 days after transfection (n=3 biologically independent experiments, mean ± SD, *****P*<0.0001, ****P*<0.001, **P*<0.05, Student's t-test).

Supplementary Fig. 7

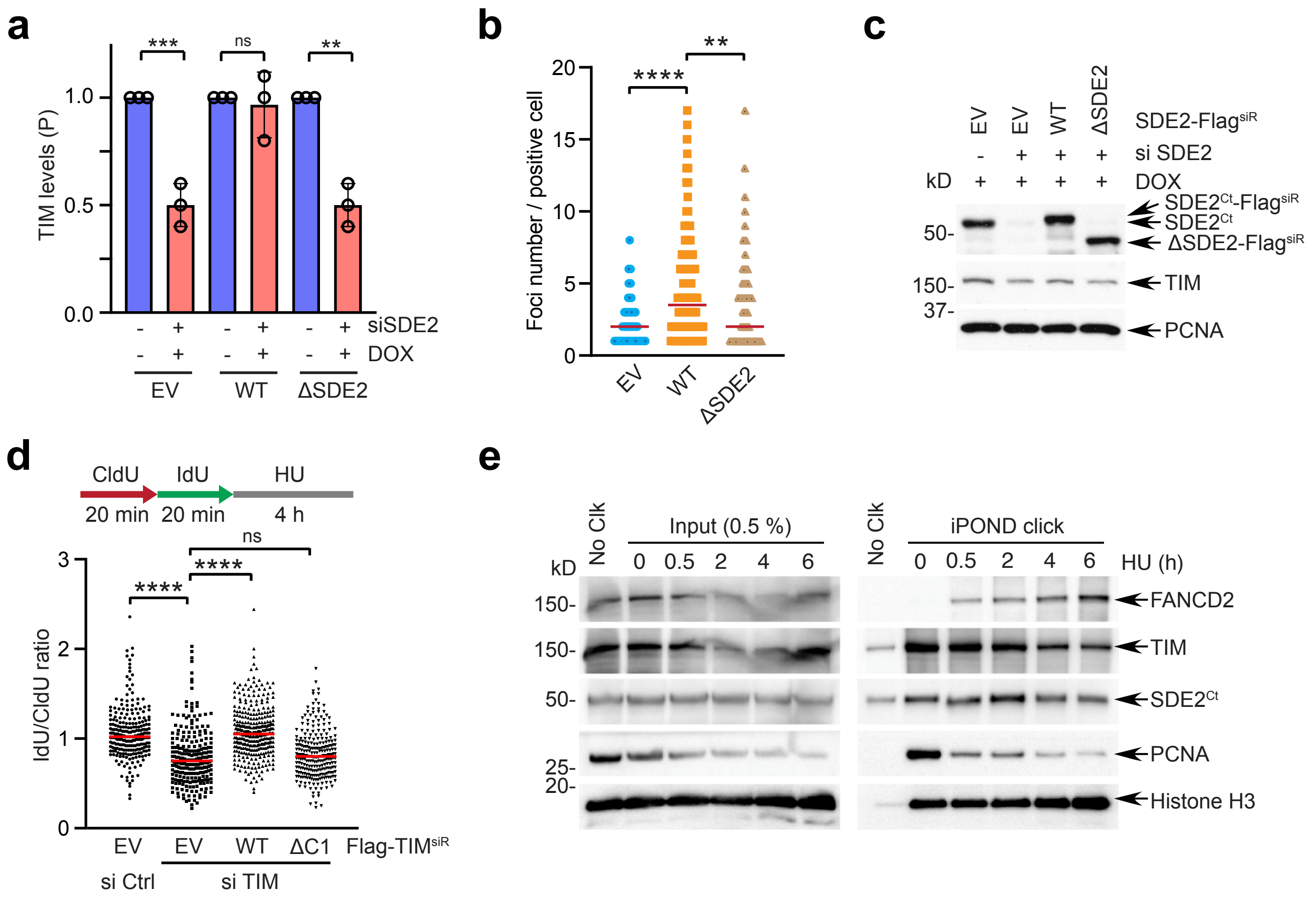

Supplementary Figure 7.
(related to Figure 7).

a Quantification of TIM levels in the P fraction from subcellular-fractionated Retro-X SDE2 WT or ΔSDE2 cells following SDE2 siRNA transfection and doxycycline (dox) induction (n=3 biologically independent experiments, mean ± SD, ****P*<0.001, ***P*<0.01, Student's t-test). **b** Quantification of TIM PLA:EdU foci numbers from the PLA-positive cells. Red bars represent median (n=3 pooled from three independent experiments, *****P*<0.0001, ***P*<0.01, Mann-Whitney test). **c** WB analysis to confirm the reconstitution of Flag-tagged SDE2 WT or ΔSDE2 mutant by dox induction following siRNA transfection in Retro-X U2OS cells used for the DNA combing analyses in Figures 7d and 7e. **d** U2OS cells knocked-down for TIM and complemented with either EV, WT or ΔC1 TIM constructs were treated with 4 mM HU for 4 h after CldU and IdU labeling. Representative dot plot of the DNA fiber IdU/CldU track length ratio from two independent experiments (>160 tracks per condition, n=2, ****P<0.0001, Mann-Whitney, n.s. not significant). **e** WB analysis of iPOND samples from EdU pulse and 2 mM HU treatment for the indicated times.

Table S1. Oligonucleotides

**siRNA target sequences**		
SDE2-1 aaACGGCAATGGCCTACTAAA	Qiagen	custom
SDE2-5 acGCAGTTATTGATAAGGAAA	Qiagen	custom
SDE2-7 ctGAATAAGGATAAAGAGACA	Qiagen	custom
TIM-1 ggGTAGCTTAGTCCTTTCAAA	Qiagen	custom
TIM-2 aaGAGCTAAGAAGCCTAGGGG	Qiagen	custom
TIPIN cgTGATTGACCTACCAGATTA	Qiagen	custom
FANCD2 ttGGAGGAGATTGATGGTCTA	Qiagen	custom
ETAA1 atTGACAAAGCAGTTAGGTAA	Ambion	Cat# s29018
TOPBP1 aaCTCACCTTATTGCAGGAGA	Ambion	Cat# s21823
SMARCAL1 caGCTTTGACCTTCTTAGCAA	Qiagen	custom
MRE11 atGAAAGGCTCTATCGAATGT	Qiagen	custom
CtIP aaCGAATCTTAGATGCACAAA	Qiagen	custom
DNA2 gcATAGCCAGTAGTATTCGAT	Qiagen	custom
EXO1 ttGCCTGAGAATAATATGTCT	Qiagen	custom
BRCA2 ttGAAGAATGCAGGTTTAATA	Qiagen	custom

**SDM Primers**	
SDE2 Δ108-150	CGGGATCTCAGTGGAAGGTTCACCAGCCCC GACTAC
SDE2 K132A/K135A	GAG CGA GAG GCT GAA gcG GAG CAG gcG CGG CTG GAG CGA CT
TIM Δ1132-1208	AAA GAG CAC CGA GCA tAA GCC CTG AGG GCC
TIM Δ882-1208	C AAG GAC TTC CAA AGG tAA GGA ACC CAT ATT G
TIM T1078D	GCC TCT GGG CAG GAA gaC TTC TGG CGA ATT CC
TIM E1049Q	TTG GTG CCA CTC ACA cAG GAA AAT GAG GAA G
TIM E1056Q	AAT GAG GAA GCC ATG cAA AAC GAA CAG TTT C

Table S2. Chemicals and Reagents

Hydroxyurea (HU)	Sigma-Aldrich	Cat# H8627
5-iodo-2´-deoxyuridine (IdU)	Sigma-Aldrich	Cat# I7125
5-chloro-2´-deoxyuridine (CldU)	Sigma-Aldrich	Cat# C6891
5-ethynyl-2´-deoxyuridine (EdU)	Thermo Fisher Scientific	Cat# A10044
5-bromo-2´-deoxyuridine (BrdU)	Sigma-Aldrich	Cat# B5002
H_2O_2	Sigma-Aldrich	Cat# H1009
RNAiMAX transfection reagent	Thermo Fisher Scientific	Cat# 13778150
Genejuice transfection reagent	MilliporeSigma	Cat# 70967
Xfect transfection reagent	Clontech Laboratories	Cat# 631317
cOmplete, EDTA-free protease inhibitor cocktail	MilliporeSigma	Cat# 11873580001
Halt phosphatase inhibitor cocktail	Thermo Fisher Scientific	Cat# 78420
MG132	Sigma-Aldrich	Cat# C2211
Cycloheximide	Sigma-Aldrich	Cat# C4859
Camptothecin	Sigma-Aldrich	Cat# C9911
Doxycycline hyclate	Sigma-Aldrich	Cat# D9891
Mirin	Sigma-Aldrich	Cat# M9948
Puromycin	Sigma-Aldrich	Cat# P8833
Biotin-phenol (BP)	R&D Systems	Cat# 6241
Thymidine	Sigma-Aldrich	Cat# T1895
Streptavidin, Alexa Fluor 594	Thermo Fisher Scientific	Cat# S11227
Streptavidin-HRP	Thermo Fisher Scientific	Cat# S911
Streptavidin agarose	Thermo Fisher Scientific	Cat# 20359
Streptavidin agarose	MilliporeSigma	Cat# 69203
Biotin azide	Thermo Fisher Scientific	Cat# B10184
Nocodazole	Sigma-Aldrich	Cat# M1404
Sodium ascorbate	VWR	Cat# 95035-692/S1349
Trolox	Sigma-Aldrich	Cat# 238813
Sodium azide	VWR	Cat# AA14314-22/14314
Aprotinin	Sigma-Aldrich	Cat# A6279
Leupeptin	Sigma-Aldrich	Cat# L2884
Ponceau S	Boston Bioproducts	Cat# ST-180
FLAG M2 affinity gel	Sigma-Aldrich	Cat# A2220
Glutathione agarose	Thermo Fisher Scientific	Cat# 16100
Dynabeads protein G	Thermo Fisher Scientific	Cat# 10003D
SYBR Gold nucleic acid gel stain	Thermo Fisher Scientific	Cat# S11494

Table S3. Antibodies

BRCA2 (Ab-1) 1:500	MilliporeSigma	Cat# OP-95
CHK1 1:1000	Santa Cruz	Cat# sc-8408
pCHK1 S345 1:1000	Cell Signaling Technology	Cat# 2341
FANCD2 (FI-17) 1:1000	Santa Cruz	Cat# sc-20022
FLAG 1:500	Sigma-Aldrich	Cat# F1804
GFP (B-2) 1:1000	Santa Cruz Biotechnology	Cat# sc-9996
GFP (polyclonal) 1:250	Abcam	Cat# ab290
γH2AX S139 1:500	Millipore	Cat# 05-536
γH2AX S139 1:500	Cell Signaling Technology	Cat# 2577
HA (6E2) 1:1000	Cell Signaling Technology	Cat# 2367
Histone H3 1:1000	Abcam	Cat# ab1791
HSC70 (B6) 1:2000	Santa Cruz	Cat# sc-7298
MCL-1 1:1000	Bethyl Laboratories	Cat# A302-715A
MCM6 (H-8) 1:1000	Santa Cruz	Cat# sc-393618
Myc (9E10) 1:1000	Santa Cruz	Cat# sc-40
ORC-2 1:1000	BD Biosciences	Cat# 551178
p97 1:1000	Cell Signaling Technology	Cat# 2648
PARP1 1:1000	Bethyl Laboratories	Cat# A301-376A-T
PARP1 (F-2) 1:1000	Santa Cruz	Cat# sc-8007
PCNA (PC-10) 1:50	Santa Cruz	Cat# sc-56
RPA32 1:1000	MilliporeSigma	Cat# MABE285
pRPA32 S4/S8 1:1000	Bethyl Laboratories	Cat# A300-245A-M
pRPA32 S33 1:1000	Bethyl Laboratories	Cat# A300-246A
SDE2 1:400	Sigma Atlas	Cat# HPA031255
SMARCAL1 1:1000	Santa Cruz	Cat# sc-376377
TIMELESS 1:500	Bethyl Laboratories	A300-961A-M
TIPIN 1:500	Bethyl Laboratories	Cat# A301-474A
γ-Tubulin 1:2000	Bethyl Laboratories	Cat# A302-631A
α-Tubulin 1:2000	Santa Cruz	Cat# sc-32293
Cyclin E (HE12) 1:1000	Santa Cruz	Cat# sc-247
Cyclin A (B-8) 1:1000	Santa Cruz	Cat# sc-271682
KU80 1:1000	Cell Signaling Technology	Cat# 2753
β-Actin 1:2000	Thermo Fisher Scientific	Cat# MA5-15739
BrdU (BU-1) 1:300	Thermo Fisher Scientific	Cat# MA3-071
BrdU (IdU) (B44) 1:5	BD Biosciences	Cat# 347580
BrdU (CldU) (BUI/75 ICR1) 1:25	Abcam	Cat# ab6326
Alexa Fluor 647 Azide	Thermo Fisher Scientific	Cat# A-10277
Goat anti-rat Alexa Fluor 594 1:100	Thermo Fisher Scientific	Cat# A-11007
Goat anti-mouse Alexa Fluor 488 1:100	Thermo Fisher Scientific	Cat# A-11001
Goat anti-rat Alexa Fluor 488 1:100	Thermo Fisher Scientific	Cat# A-11006
Goat anti-mouse Alexa Fluor 568 1:100	Thermo Fisher Scientific	Cat# A-11004

Anti-ssDNA, clone 16-19 1:100	EMD Millipore	Cat# MAB3034
Goat anti-mouse Alexa Fluor 647 1:100	Thermo Fisher Scientific	Cat# A-21241
Biotin (mouse) 1:2000	Jackson ImmunoResearch	Cat# 200-002-211
Biotin (rabbit) 1:3000	Bethyl Laboratories	Cat# A150-109A
Trueblot Ultra: anti-mouse IgG HRP 1:3000	Rockland	Cat# 18-8817-33
Light-chain specific anti-rabbit IgG HRP 1:3000	Jackson ImmunoResearch	Cat# 211-032-171
Normal Rabbit IgG	Millipore-Sigma	Cat# 12-370
