# Supplementary material for: SDE2 integrates into the TIMELESS-TIPIN complex to protect stalled replication forks
Source: Nat Commun. 2020 Oct 30;11:5495. doi: 10.1038/s41467-020-19162-5 (PMC7603486; doi:10.1038/s41467-020-19162-5)
Supplement: Supplementary file 3 — Reporting Summary [file 41467_2020_19162_MOESM3_ESM.pdf]

nature research

Corresponding author(s): Hyungjin Kim, Jung-Eun Yeo

Last updated by author(s): Sep 20, 2020

Reporting Summary

Nature Research wishes to improve the reproducibility of the work that we publish. This form provides structure for consistency and transparency in reporting. For further information on Nature Research policies, see our Editorial Policies and the Editorial Policy Checklist.

Statistics

For all statistical analyses, confirm that the following items are present in the figure legend, table legend, main text, or Methods section.

n/a	Confirmed	
☐	☒	The exact sample size (*n*) for each experimental group/condition, given as a discrete number and unit of measurement
☐	☒	A statement on whether measurements were taken from distinct samples or whether the same sample was measured repeatedly
☐	☒	The statistical test(s) used AND whether they are one- or two-sided<br>*Only common tests should be described solely by name; describe more complex techniques in the Methods section.*
☒	☐	A description of all covariates tested
☒	☐	A description of any assumptions or corrections, such as tests of normality and adjustment for multiple comparisons
☐	☒	A full description of the statistical parameters including central tendency (e.g. means) or other basic estimates (e.g. regression coefficient) AND variation (e.g. standard deviation) or associated estimates of uncertainty (e.g. confidence intervals)
☐	☒	For null hypothesis testing, the test statistic (e.g. *F*, *t*, *r*) with confidence intervals, effect sizes, degrees of freedom and *P* value noted<br>*Give P values as exact values whenever suitable.*
☒	☐	For Bayesian analysis, information on the choice of priors and Markov chain Monte Carlo settings
☒	☐	For hierarchical and complex designs, identification of the appropriate level for tests and full reporting of outcomes
☒	☐	Estimates of effect sizes (e.g. Cohen's *d*, Pearson's *r*), indicating how they were calculated

Our web collection on statistics for biologists contains articles on many of the points above.

Software and code

Policy information about availability of computer code

Data collection	NIS-Elements, Research BR software ver.4; Attune NxT software v2.7
Data analysis	NIS-Elements, Research BR software ver.4; Attune NxT software v2.7; Fiji-Image J, image analysis software 2.0.0-rc-69/1.52p; GraphPad Prism 8 GraphPad Software; Adobe Photoshop CC/CS6

For manuscripts utilizing custom algorithms or software that are central to the research but not yet described in published literature, software must be made available to editors and reviewers. We strongly encourage code deposition in a community repository (e.g. GitHub). See the Nature Research guidelines for submitting code & software for further information.

Data

Policy information about availability of data

All manuscripts must include a data availability statement. This statement should provide the following information, where applicable:

- Accession codes, unique identifiers, or web links for publicly available datasets
- A list of figures that have associated raw data
- A description of any restrictions on data availability

The data that support the findings of this study are available within the Article, Supplementary Information, or from the corresponding author upon reasonable request. The source data underlying Figs. 1c, 1e, 1h, 2a-h, 3a-g, 4a-f, 5a-g, 6a-h, 7a-e and Supplementary Figs. 1a-d, 2a-e, 3a-f, 4a-d, 4f, 5a-e, 6b-h, 7a-e are provided as a Source Data file.

Field-specific reporting

Please select the one below that is the best fit for your research. If you are not sure, read the appropriate sections before making your selection.

☒ Life sciences ☐ Behavioural & social sciences ☐ Ecological, evolutionary & environmental sciences

For a reference copy of the document with all sections, see nature.com/documents/nr-reporting-summary-flat.pdf

Life sciences study design

All studies must disclose on these points even when the disclosure is negative.

Sample size	No statistical method was considered to predetermine sample size. For independent biological replicates, individual replicate numbers are given in figure legends. Sample size was determined based on relevant literature and previous experiments. For the DNA combing assay, 100-300 DNA fibers were analyzed per experimental condition, which is widely accepted in the field (Quinet A, Carvajal-Maldonado D, Lemacon D, Vindigni A. DNA Fiber Analysis: Mind the Gap!. Methods Enzymol. 2017;591:55-82. doi:10.1016/bs.mie.2017.03.019, PMID: 28645379; Técher H, Koundrioukoff S, Azar D, et al. Replication dynamics: biases and robustness of DNA fiber analysis. J Mol Biol. 2013;425(23):4845-4855. doi:10.1016/j.jmb.2013.03.040, PMID: 23557832).
Data exclusions	No data were excluded.
Replication	All cellular and biochemical experiments were repeated as independent biological replicates. Western blotting, DNA combing, immunofluorescence, DNA comet assay, survival assay, and cell cycle analysis were performed at least twice independently, mostly three times. Survival assays were technically duplicated in each set. We confirmed that the repeated experiments showed similar results and all attempts to reproduce results were successful.
Randomization	To set up exponentially growing cells for experiments, including drug treatment, we split a dish of trypsinized cells equally and allocated randomly to each experimental group to perform Western blotting, DNA combing, immunofluorescence, DNA comet assay, survival assay, cell cycle analysis, and other cellular assays mentioned in the methods section. Other than that, randomization was not needed for the study as this work does not involve particular animals or groups.
Blinding	For Western blotting, DNA combing, immunofluorescence, DNA comet assay, survival assay, and cell cycle analysis, data of different groups were collected while no blinding was used since blinding was not relevant for this kind of experiments in which researchers are aware of particular experimental conditions. When data were analyzed by software, researchers were generally not blinded to group allocation.

Reporting for specific materials, systems and methods

We require information from authors about some types of materials, experimental systems and methods used in many studies. Here, indicate whether each material, system or method listed is relevant to your study. If you are not sure if a list item applies to your research, read the appropriate section before selecting a response.

Materials & experimental systems

n/a	Involved in the study	
☐	☒	Antibodies
☐	☒	Eukaryotic cell lines
☒	☐	Palaeontology and archaeology
☒	☐	Animals and other organisms
☒	☐	Human research participants
☒	☐	Clinical data
☒	☐	Dual use research of concern

Methods

n/a	Involved in the study	
☒	☐	ChIP-seq
☐	☒	Flow cytometry
☒	☐	MRI-based neuroimaging

Antibodies

Antibodies used

BRCA2 (Ab-1) 1:500 MilliporeSigma Cat# OP-95
CHK1 1:1000 Santa Cruz Cat# sc-8408
pCHK1 S345 1:1000 Cell Signaling Technology Cat# 2341
FANCD2 (FI-17) 1:1000 Santa Cruz Cat# sc-20022
FLAG 1:500 Sigma-Aldrich Cat# F1804
GFP (B-2) 1:1000 Santa Cruz Biotechnology Cat# sc-9996
GFP (polyclonal) 1:250 Abcam Cat# ab290
γH2AX S139 1:500 Millipore Cat# 05-536
γH2AX S139 1:500 Cell Signaling Technology Cat# 2577
HA (6E2) 1:1000 Cell Signaling Technology Cat# 2367
Histone H3 1:1000 Abcam Cat# ab1791
HSC70 (B6) 1:2000 Santa Cruz Cat# sc-7298

MCL-1 1:1000 Bethyl Laboratories Cat# A302-715A
MCM6 (H-8) 1:1000 Santa Cruz Cat# sc-393618
Myc (9E10) 1:1000 Santa Cruz Cat# sc-40
ORC-2 1:1000 BD Biosciences Cat# 551178
p97 1:1000 Cell Signaling Technology Cat# 2648
PARP1 1:1000 Bethyl Laboratories Cat# A301-376A-T
PARP1 (F-2) 1:1000 Santa Cruz Cat# sc-8007
PCNA (PC-10) 1:50 Santa Cruz Cat# sc-56
RPA32 1:1000 MilliporeSigma Cat# MABE285
pRPA32 S4/S8 1:1000 Bethyl Laboratories Cat# A300-245A-M
pRPA32 S33 1:1000 Bethyl Laboratories Cat# A300-246A
SDE2 1:400 Sigma Atlas Cat# HPA031255
SMARCAL1 1:1000 Santa Cruz Cat# sc-376377
TIMELESS 1:500 Bethyl Laboratories A300-961A-M
TIPIN 1:500 Bethyl Laboratories Cat# A301-474A
γ-Tubulin 1:2000 Bethyl Laboratories Cat# A302-631A
α-Tubulin 1:2000 Santa Cruz Cat# sc-32293
Cyclin E (HE12) 1:1000 Santa Cruz Cat# sc-247
Cyclin A (B-8) 1:1000 Santa Cruz Cat# sc-271682
KU80 1:1000 Cell Signaling Technology Cat# 2753
β-Actin 1:2000 Thermo Fisher Scientific Cat# MA5-15739
BrdU (BU-1) 1:300 Thermo Fisher Scientific Cat# MA3-071
BrdU (IdU) (B44) 1:5 BD Biosciences Cat# 347580
BrdU (CldU) (BUI/75 ICR1) 1:25 Abcam Cat# ab6326
Alexa Fluor 647 Azide Thermo Fisher Scientific Cat# A-10277
Goat anti-rat Alexa Fluor 594 1:100 Thermo Fisher Scientific Cat# A-11007
Goat anti-mouse Alexa Fluor 488 1:100 Thermo Fisher Scientific Cat# A-11001
Goat anti-rat Alexa Fluor 488 1:100 Thermo Fisher Scientific Cat# A-11006
Goat anti-mouse Alexa Fluor 568 1:100 Thermo Fisher Scientific Cat# A-11004
Anti-ssDNA, clone 16-19 1:100 EMD Millipore Cat# MAB3034
Goat anti-mouse Alexa Fluor 647 1:100 Thermo Fisher Scientific Cat# A-21241
Biotin (mouse) 1:2000 Jackson ImmunoResearch Cat# 200-002-211
Biotin (rabbit) 1:3000 Bethyl Laboratories Cat# A150-109A
Trueblot Ultra: anti-mouse IgG HRP 1:3000 Rockland Cat# 18-8817-33
Light-chain specific anti-rabbit IgG HRP 1:3000 Jackson ImmunoResearch Cat# 211-032-171
Normal Rabbit IgG Millipore-Sigma Cat# 12-370

Validation

All antibodies were used as per manufacturer's recommendations. Antibodies were validated by siRNA or cDNA transfection.

Eukaryotic cell lines

Policy information about cell lines

Cell line source(s)

U2OS, 293T, and BJ-TERT cells were obtained from ATCC.

Authentication

The ATCC used SRT and isoenzyme profiles for cell line authentication. Additional authentication in the laboratory was performed regularly based on morphology and gene/protein expression.

Mycoplasma contamination

Cell lines were periodically tested for mycoplasma contamination (except BJ-TERT cells) and confirmed negative.

Commonly misidentified lines
(See ICLAC register)

No commonly misidentified cell lines were used in this study.

Flow Cytometry

Plots

Confirm that:

☒ The axis labels state the marker and fluorochrome used (e.g. CD4-FITC).

☒ The axis scales are clearly visible. Include numbers along axes only for bottom left plot of group (a 'group' is an analysis of identical markers).

☒ All plots are contour plots with outliers or pseudocolor plots.

☒ A numerical value for number of cells or percentage (with statistics) is provided.

Methodology

Sample preparation

To label replicating cells, siRNA-transfected cells were incubated with 10 μM EdU (Thermo Fisher) for 30 min before harvest. Harvested cells were fixed with 4% paraformaldehyde for 15 min at RT, permeabilized by saponin-based permeabilization buffer (Thermo Fisher) for 15 min, and subjected to EdU-click reaction using Alexa Fluor 488 picolyl azide and click-iT Plus EdU flow cytometry assay kit (Thermo Fisher) following manufacturer’s protocol. Cells were washed once and resuspended

with 200 µg/mL PureLinkTM RNase A and eBioscienceTM 7-AAD viability staining solution (Thermo Fisher). After 30 min of incubation at 37 °C, cells were analyzed using Attune NxT acoustic focusing cytometer.

Instrument	Attune NxT acoustic focusing cytometer, Thermo Fisher
Software	Attune NxT software v2.7, Thermo Fisher
Cell population abundance	More than 10,000 live cells were collected to ensure the quality of samples per event.
Gating strategy	All samples were gated for FSC-H/SSC-H to exclude dead cells and debris before cell cycle analysis. Gates were drawn from distinct and observable stained populations. An example of FSC-H/SSC-H gating to specify live cells is shown in the Source Data file, which is applicable to all figures regardless of whether cells were stained or not. Unstained cells were used to establish boundaries and draw the positive populations from the negative.

☒ Tick this box to confirm that a figure exemplifying the gating strategy is provided in the Supplementary Information.
